# Role of inflammation in a rat model of radiation retinopathy

**DOI:** 10.1186/s12974-024-03151-2

**Published:** 2024-06-24

**Authors:** Cécile Lebon, Denis Malaise, Nicolas Rimbert, Manon Billet, Gabriel Ramasamy, Jérémie Villaret, Frédéric Pouzoulet, Alexandre Matet, Francine Behar-Cohen

**Affiliations:** 1 Centre de Recherche des Cordeliers, INSERM UMRS 1138, Sorbonne Université, Physiopathology of Ocular Diseases: Therapeutic Innovations, 15 rue de l’école de Médecine, Paris, 75006 France; 2 Ocular Oncology Department, Institut Curie, 26 rue d’Ulm, Paris, 75005 France; 3 Translational Research Department, Experimental Radiotherapy Platform, Institut Curie, Université Paris Saclay, 15 Rue Georges Clemenceau, Orsay, 91400 France; 4 Centre Hospitalier National d’Ophtalmologie des Quinze-Vingts, Paris, 75012 France; 5 Centre Rétine Gallien, Bordeaux, 33000 France; 6 Université Paris Cité, 15 rue de l’école de Médecine, Paris, 75006 France; 7 Assistance Publique, Hôpitaux de Paris, Hôpital Cochin, 27 rue du Faubourg Saint-Jacques, Ophtalmopole, Paris, 75014 France

**Keywords:** Radiation retinopathy, Microglia, Inflammation, Macrophages, Blood retinal barrier, Microangiopathy, Hypoxia, Retinal pigment epithelium

## Abstract

Radiation retinopathy (RR) is a major side effect of ocular tumor treatment by plaque brachytherapy or proton beam therapy. RR manifests as delayed and progressive microvasculopathy, ischemia and macular edema, ultimately leading to vision loss, neovascular glaucoma, and, in extreme cases, secondary enucleation. Intravitreal anti-VEGF agents, steroids and laser photocoagulation have limited effects on RR. The role of retinal inflammation and its contribution to the microvascular damage occurring in RR remain incompletely understood. To explore cellular and vascular events after irradiation, we analyzed their time course at 1 week, 1 month and 6 months after rat eyes received 45 Gy X-beam photons. Müller glial cells, astrocytes and microglia were rapidly activated, and these markers of retinal inflammation persisted for 6 months after irradiation. This was accompanied by early cell death in the outer retina, which persisted at later time points, leading to retinal thinning. A delayed loss of small retinal capillaries and retinal hypoxia were observed after 6 months, indicating inner blood‒retinal barrier (BRB) alteration but without cell death in the inner retina. Moreover, activated microglial cells invaded the entire retina and surrounded retinal vessels, suggesting the role of inflammation in vascular alteration and in retinal cell death. Radiation also triggered early and persistent invasion of the retinal pigment epithelium by microglia and macrophages, contributing to outer BRB disruption. This study highlights the role of progressive and long-lasting inflammatory mechanisms in RR development and demonstrates the relevance of this rat model to investigate human pathology.

**Supplementary Information:**

The online version contains supplementary material available at 10.1186/s12974-024-03151-2.

## Introduction

Radiation retinopathy (RR) defines the retinal changes that occur secondary to ocular exposure to any source of radiation. Its most frequent cause is irradiation to treat ocular tumors, such as plaque brachytherapy or proton beam therapy for uveal melanomas, or collateral irradiation for orbital, facial or cerebral tumors. Clinical signs of RR are delayed and are usually observed from 6 months to 3 years after irradiation and most commonly after 2 years. Patients present progressive vasculopathy with microvascular occlusion and telangiectasia, areas of non-perfusion and subsequent proliferative complications that can cause retinal hemorrhages and neovascular glaucoma, which might require the removal of the eye as a last resort. As time progresses, retinal layer thinning can be associated with visual impairment. Radiation macular edema, a frequent sign of RR, usually precedes any ischemic retinal changes resulting from areas of capillary leakage [1,2].

RR onset and severity are closely associated with the cumulative radiation dose, dose fractioning, and extent of exposure. The threshold for complications is approximately 30–35 Gy [3,4]. Dose fractionation and localized delivery can limit RR, which still develops in a large proportion of irradiated patients in the absence of recognized prophylaxis and limited therapeutic options, mostly relying on intravitreal anti-VEGF (vascular endothelial growth factor) and laser photocoagulation, which delay RR but do not prevent it [5]. RR thus remains an unmet medical need.

The exact mechanisms leading to RR are not fully understood. It has been postulated that radiation first alters endothelial cells in retinal capillaries, leading to their death during the subclinical acute phase [6,7]. Other endothelial cells subsequently die while attempting to divide due to radiation-induced DNA damage. Focal endothelial cell loss can lead to microvascular aneurysms, occlusions, vessel abnormalities and vascular damage, which manifests as areas of non-perfusion and vascular leakage [6]. The slow turnover of endothelial cells could explain the late onset and progressive development of RR. The role of retinal inflammation has been characterized in several retinal disorders, including diabetic retinopathy [8–10] and age-related macular degeneration (AMD) [11–13], but retinal inflammation after radiation has scarcely been explored, although clinical observations suggest that anti-inflammatory drugs such as corticosteroids may reduce RR manifestations [14]. In addition, the time course of glial and inflammatory cell activation has not been fully described, despite leukostasis being identified as an early pathogenic event [15], and whether retinal inflammation contributes to the microvascular damage occurring in RR remains uncertain. To address this issue, we developed a preclinical model of RR in rats and analyzed the time course of cellular and vascular events. Our results indicate that after irradiation, retinal inflammation progresses and intensifies and could play a pathogenic role in irreversible retinal and vascular damage.

## Materials and methods

### Animals

Six-week-old Long-Evans male rats (Janvier Labs, France) were used in this study. All experimental procedures were performed in accordance with the Association for Research in Vision and Ophthalmology (ARVO) statement for the use of animals in ophthalmic and vision research. All animal experiments were conducted following European animal welfare and ethical guidelines and were approved by the French government (authorization no. 34397-2021121621111587). The animals were housed in our animal facility, which is accredited for performing experiments on rodents, under a 12 h light/dark cycle with free access to standardized pelleted food and water.

After one week of acclimatization, the animals were irradiated in the Small Animal Radiation Research Platform (SARRP, Xstrahl, USA) under gaseous anesthesia (5% isoflurane for induction and 3% isoflurane for maintenance).

The photon irradiations were realized with a positioning scanner and a set of specific collimators to accurately target the rat’s bulging eyeball while avoiding the irradiation of organs at risk (lacrimal gland, digestive tractus, brain). The device’s irradiation parameters were 220 kV and 13 mA, with an average X-ray tube energy of 75 keV and a half-value layer (HVL) of 0.89. The irradiation field size was 5 × 5 mm. The dose distribution to the eyeball was calculated using the “MuriPlan” treatment plan software (Xstrahl, Walsall Wood, UK). One eye per animal was irradiated for 3 consecutive days with a 15 Gy dose per day, leading to a total dose of 45 Gy. The contralateral eye was used as a control. The animals were then kept in the animal facility for 1 week, 1 month or 6 months after photon irradiation for analysis.

### Evaluation of retinal hypoxia and retinal vessels

Retinal perfusion and hypoxia were evaluated using a Hypoxyprobe-1 kit (Hypoxyprobe, Inc., MA, USA). This kit contains pimonidazole, a probe classically used to detect hypoxic area. Three hours prior to sacrifice, the rats received an intraperitoneal injection of pimonidazole (60 mg/kg body weight). The rats were sacrificed, and the enucleated eyes were fixed for 30 min in 4% paraformaldehyde (PFA). The eyes were then dissected, and the retinas were flat-mounted, fixed for 10 min in acetone at -20 °C, blocked with 10% fetal bovine serum in phosphate-buffered saline (PBS, Life Technologies, MA, USA) supplemented with 0.1% Triton for 30 min and incubated with an anti-pimonidazole antibody at a 1:100 dilution in blocking solution overnight. The retinas were then rinsed and incubated with secondary antibody (Alexa Fluor-conjugated 546 anti-mouse, Invitrogen, USA) together with FITC-conjugated lectin (Sigma Aldrich) at 1:200 for 2 h at room temperature. Flat mounts were mounted with fluorescent aqueous mounting medium (Dako Ltd., UK). Images were acquired with a confocal microscope (LSM 710 Carl Zeiss, France), mostly mosaics with Z stacks, and measurements were performed using ImageJ software with a macro tool developed on Fiji by the CHIC (Centre d’histologie, d’imagerie et de cytométrie, Centre de recherche des Cordeliers UMR S 1138, Paris, France). Statistics were performed on Prism 8 software (GraphPad, MA, USA), using one-way ANOVA statistical tests followed by Tukey’s multiple comparison test, *n* ≥ 5 eyes.

### Retina and RPE flat mounts

The rats were sacrificed, and the enucleated eyes were fixed for 30 min in 4% PFA. Eyes were then dissected, and both the retinas and retinal pigment epithelium/choroid complexes were flat-mounted, fixed for 10 min in acetone at -20 °C, and blocked with 10% fetal bovine serum in PBS supplemented with Triton 0, 1% for 30 min. The flat mounts were incubated with primary antibody at a dilution of 1:100 (anti-podocalyxin #MAB1556, R&D Systems, MN, USA; anti-Iba1 #019-19741 FUJIFILM Wako Chemicals, VA, USA; anti-CD68 #sc-59103, Santa Cruz, TX, USA; anti-ZO-1 #40-2200, Invitrogen, MA, USA) in blocking solution for 4 days at 4 °C. After rinsing, the flat mounts were incubated with the appropriate secondary antibody at 1:200 (Molecular Probes Alexa Fluor, Invitrogen, MA, USA). RPE flat mounts were then incubated with phalloidin (Life Technologies) at a dilution of 1:300 for 1 h. Flat mounts were incubated with 4’,6-diamidino-2-phenylindole (DAPI) at a dilution of 1:5000 for 5 min under constant agitation. They were finally mounted with fluorescent aqueous mounting medium (Dako Ltd., UK). Images were acquired with a confocal microscope (LSM 710 Carl Zeiss, France), and image analysis of RPE flat mounts was performed using a macro tool developed on Fiji software. Statistics were performed on Prism 8 software (GraphPad, MA, USA), using one-way ANOVA statistical tests followed by a Dunnett’s multiple comparison tests for RPE cell surface and circularity measurements; and Chi-square test to compare RPE cell area distribution. 7255 cells were analyzed for control RPE, 7283 cells 1 month after irradiation, and 6898 cells 6 months after irradiation.

We performed 3D reconstructions of z-stack flat mounts using IMARIS (Oxford Instrument, UK).

### Cryosections

After euthanasia, eyes were enucleated, mounted in Tissue-Tek OCT and frozen in liquid nitrogen. 10 μm-thick cryosections were cut on a microtome (Leica CM3050S, France). A TUNEL assay was performed to evaluate cell death within the retina. The sections were first fixed for 15 min in 4% PFA and permeabilized for 15 min in 0.3% Triton X-100/PBS. The TUNEL assay was performed according to the manufacturer’s instructions (TMR red protocol, Roche Diagnostics, Germany). For some sections, the TUNEL assay was preceded by a dephosphorylation step according to a protocol described previously [16]. Briefly, sections were dephosphorylated with 10 U of calf intestinal alkaline phosphatase (CIAP, Invitrogen) in the associated buffer for 30 min at 37 °C and washed. Nuclei were stained by a 5 min incubation with DAPI at 1:5000 in PBS, and sections were mounted with fluorescent aqueous mounting medium (Dako Ltd., UK).

For GFAP staining, sections were washed in Ca^2+^/Mg^2+^-supplemented PBS, incubated in 4% PFA in PBS during 15 min for fixation, and washed with PBS. Sections were then incubated in 0.3% Triton X-100/PBS during 30 min for permeabilization and washed with PBS. Saturation was performed with 1% nonfat milk in PBS for 45 min. Sections were then incubated for 1 h with primary antibody (GFAP, Sigma-Merck, Germany) in 0.1% nonfat milk in PBS. After several washes, the samples were incubated with the secondary antibody in PBS for 1 h in a dark chamber. Nuclei were stained by a 5 min incubation with DAPI at 1:5000 in PBS, and sections were mounted with fluorescent aqueous mounting medium (Dako Ltd, UK). Images were acquired with a fluorescent U-25ND25 Olympus microscope, and measurements were performed with ImageJ software. TUNEL-positive cells were manually counted twice on cryosections close to the optic nerve. Statistics were performed on Prism 8 software (GraphPad, MA, USA), using Kruskal-Wallis statistical tests followed by Dunn’s multiple comparison test, *n* = 5 eyes per time point.

### Western blotting

After enucleation, neuroretinas and RPE/choroid complexes were dissected, and proteins were extracted in lysis buffer (M-Per supplemented with anti-proteases and anti-phosphatases; Thermo Fisher Scientific, MA, USA) with a pellet pestle motor on ice and centrifuged for 10 min at 15,000 rpm. Protein concentrations were calculated using a BCA protein assay (Thermo Fisher Scientific, MA, USA). Western blotting was performed with precast gels (4–12% Bis-Tris, Bolt, Thermo Fisher Scientific, MA, USA), a migration tank system (Thermo Fisher Scientific, MA, USA), a tank transfer system (Bio-Rad, CA, USA) and a nitrocellulose membrane. Red Ponceau staining was performed immediately after protein transfer. Nonspecific binding was blocked with 5% nonfat dry milk in PBS. The membranes were then incubated overnight at 4 °C with the primary antibody at a dilution of 1:1000 in PBST (0.1% Tween 20). After rinsing, the membranes were incubated with horseradish peroxidase–conjugated secondary antibody for 1 h (Vector Laboratories, Eurobio, France). Protein bands were visualized with Supersignal Pico or Femto Chemoluminescent Substrate (Thermo Fisher Scientific, MA, USA) using the iBright Imaging System (Thermo Fisher Scientific, MA, USA). FIJI software was used to quantify the integrated density of the specific band, and protein expression was reported on Ponceau staining. Statistics were performed on Prism 8 software (GraphPad, MA, USA), using Mann-Whitney statistical tests, with *n* = 4 (M1) or *n* = 5 (W1, M6).

## Results

### Development of the radiation retinopathy rat model

**Fig. 1:**
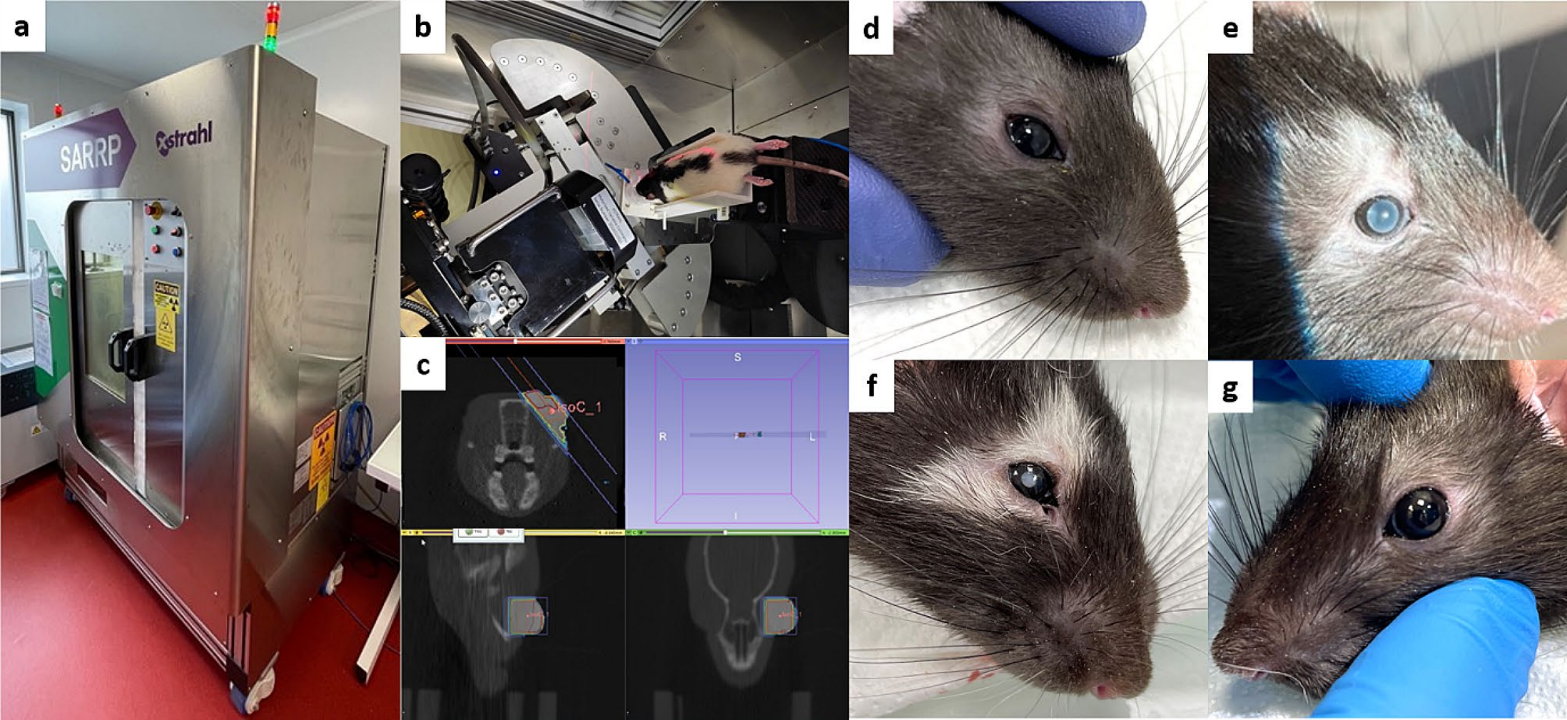
Photon irradiation animal model. Animals were irradiated into a SARRP under gaseous anesthesia (**a**,**b**). A positioning scanner prior to the photon X-beam irradiation was performed for every animal to adjust the trajectory strictly on the eyeball (**c**) whilst avoiding peripheral tissues. Irradiated eyes analyzed after one week (**d**) did not show any visible modification while hair loss, skin depigmentation, and moderate cataract were observed one month after irradiation (**e**). After six months the depigmentation was still visible, and the cataract was denser (**f**). Left eyes did not present any visible alteration six months after irradiation (**g**)

Using a SARRP device (Small Animal Radiation Research Platform) (Fig. 1a and b), a total dose of 45 Gy X-beam photons delivered over 3 days in 3 daily doses of 15 Gy was chosen among the other irradiation regimens. The bulging right eye was held and visible on the positioning scanner made just before the radiation session (Fig. 1c). As the photon X-beam trajectory was calculated and adjusted for every animal to minimize irradiation of the lachrymal gland, the brain, and other adjacent structures, after one week, the irradiated eye did not show any significant damage except for mild periocular hair loss (Fig. 1d). One month after irradiation, hair and lashes loss, skin and hair depigmentation were visible in the irradiated area, and moderate cataracts were observed in the irradiated eye (Fig. 1e). Six months after irradiation, skin and hair modifications were still present, but the cataracts were more advanced (Fig. 1f). In and around the non-irradiated left eye, the lens remained clear, and no skin or hair modifications were visible (Fig. 1g). It is important to note that no change in animal behavior was observed after photon X-beam irradiation. In vivo analyses were planned to follow the kinetics of retinal anatomic and functional changes, but the premature cataract development did not allow to perform fundus photography, OCT (Optical Coherence Tomography), angiography or ERG (electroretinography) later than 3 weeks after irradiation. However, we did not observe any significant anatomic or functional changes between prior to photon irradiation and 2 weeks after (data not shown).

### Delayed hypoxia takes place in the irradiated retina

**Fig. 2:**
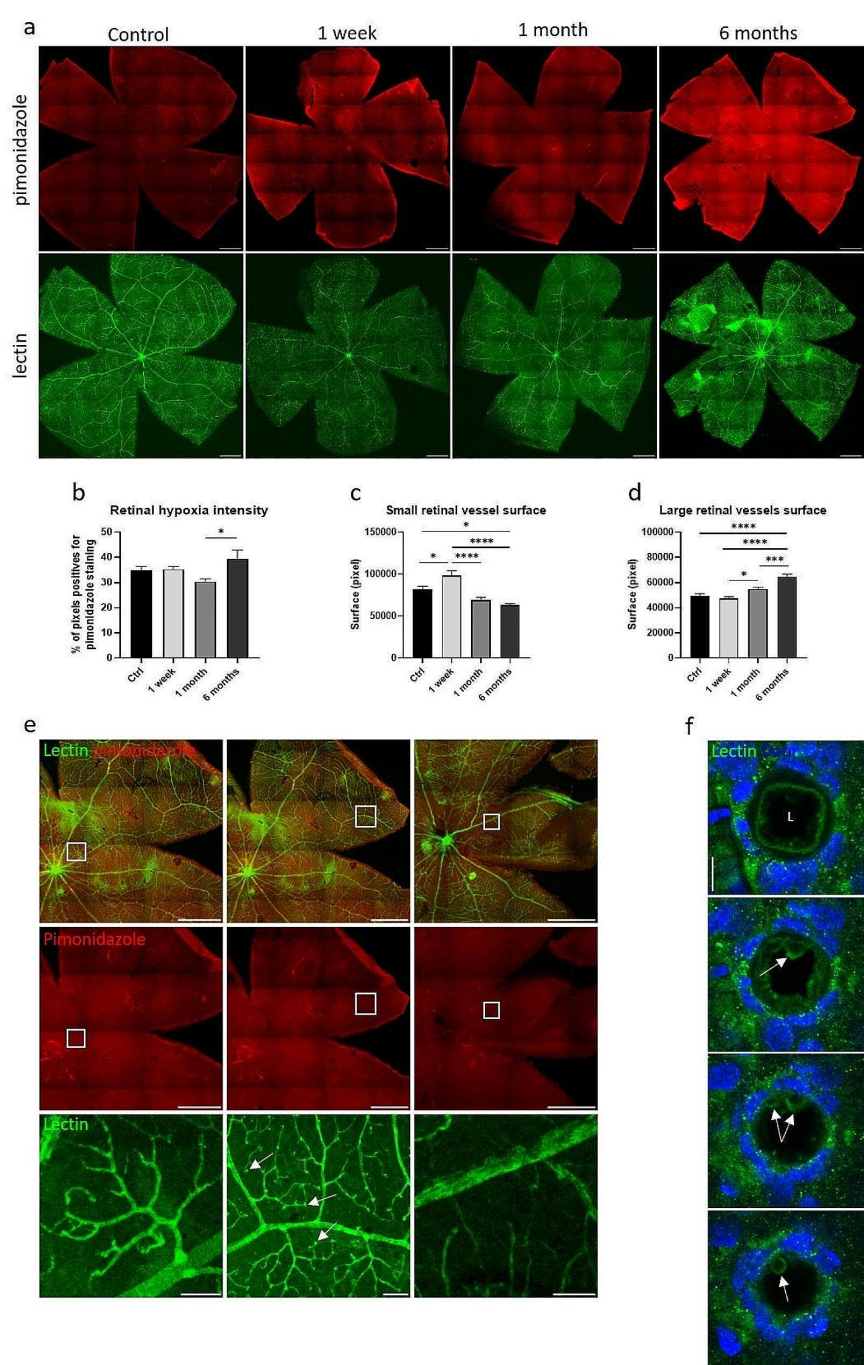
Retinal hypoxia and vascularization modifications after irradiation. (**a**) Hypoxic retinal areas are detected by the pimonidazole probe (in red) and the retinal vascularization is stained with lectin-FITC (in green) on rat flat-mounted retinas at one week, one month and six months after irradiation. Non-irradiated eyes were used as controls. Scale bars represent 1 mm. (**b**) Quantification of the retinal hypoxia intensity using the pimonidazole staining. One-way ANOVA statistical test followed by Tukey’s multiple comparison test, *n* ≥ 5 eyes. (**c**, **d**) Quantification of the surface covered by small and large vessels based on the lectin staining with a dedicated macro. After irradiation, we observed a significant and progressive decrease of small vessels and capillaries after irradiation along with a significant increase of larger vessels. One-way ANOVA statistical test followed by Tukey’s multiple comparison test, *n* ≥ 5, 13 images per flat-mounted retina were analyzed. * means *p* < 0.05; *** *p* < 0.0005; ****p < 0.0001. (**e**) Flat-mounted rat retinas stained with pimodinazole probe (in red) to lectin-FITC (in green) 6 months after irradiation. White squares show area enlarged at the bottom line. White arrows point the retinal microaneurysms. Scale bars represent 1 mm on merged and pimonidazole pictures, 100 μm on enlargements. (**f**) Transverse view of vessel blebbing in retina 6 months after irradiation. Vessel walls are stained with lectin in green and nuclei with Dapi in blue. All images represent the same vessel at different depth. L means lumen and white arrows show membrane blebs. Scale bar represents 10 μm

Local retinal hypoxia is a common feature observed in RR patients due to microvascular abnormalities and closure. Pimonidazole, a commonly used probe for detecting hypoxic areas in tissues^17,]^ was used to quantify the area of the hypoxic retina. As shown in Fig. 2, we observed a significant increase in retinal hypoxia between 1 and 6 months after irradiation, while no significant differences were detected at 1 week or 1 month after irradiation compared to non-irradiated eyes (Fig. 2a and b). It is to note that pimonidazole was evenly observed in the entire thickness of the retina after irradiation while it was mainly observed on the inner surface of the retina in controls (supplementary data).

The density and size of lectin-stained vessels also appeared to be modified (Fig. 2a and e). Indeed, quantification of the surface occupied by small or large vessels showed a first increase of small capillaries at 1 week due to vasodilation, followed by a progressive loss of small capillaries at 1 month and 6 months, mirrored by an increase of the surface occupied by larger vessels (Fig. 2c and d). Some microaneurysms were also visible at 6 months (Fig. 2e), as well as blebbing of endothelial cells at the luminal vessel membrane (Fig. 2f). A delayed alteration of retinal microvessels and subsequent hypoxia are thus observed after irradiation, similar to human radiation retinopathy.

### Retinal cell death is maintained over time in the outer retina

**Fig. 3:**
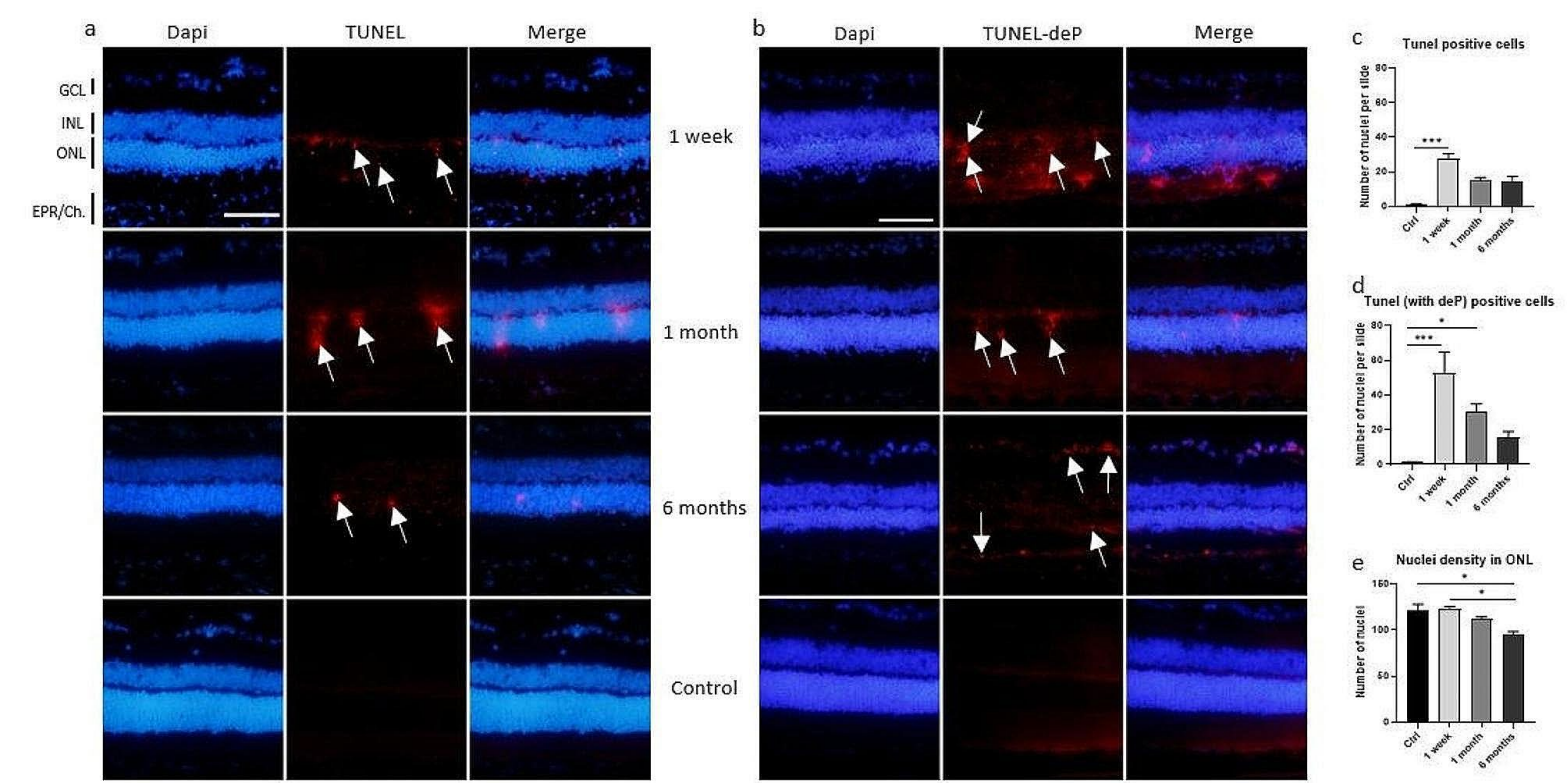
Photon irradiation induced different types of cell death in retina. (**a**) The TUNEL technique (in red) allowed the detection of caspase-dependent apoptotic nuclei (white arrows) on cryosections of irradiated eyes 1 week, 1 month and 6 months after photon irradiation or non-irradiated eyes used as control (Ctrl). Dapi was used to stain nuclei. The scale bar represents 50 μm. GCL means ganglion cells layer; INL, inner nuclear layer; ONL, outer nuclear layer; RPE/Ch, Retinal Pigment Epithelium/Choroid. Irradiation induced apoptotic death in the ONL that was quantified on (**c**), Kruskal-Wallis statistical test followed by Dunn’s multiple comparison test, *n* = 5 eyes. (**b**) The TUNEL staining in red has been preceded by a dephosphorylation step, allowing the detection of apoptotic nuclei through caspase-dependent and -independent apoptosis. More dying nuclei were visible (white arrows) after irradiation than with TUNEL alone. Nuclei were stained with DAPI. The scale bar represents 50 μm, the same scale was used for all pictures. Quantification of the number of TUNEL-positive cells was made on (**d**) with Kruskal-Wallis statistical test followed by Dunn’s multiple comparison test, *n* = 5. (**e**) We observed a progressive decrease in the nuclei density in the ONL due to irradiation, quantified by counting the number of nuclei on a constant surface (320*120 pixels), Kruskal-Wallis statistical test followed by Dunn’s multiple comparison test, *n* = 5, * means *p* < 0.05; ***p < 0.0005

We used the widely used TUNEL technique to detect caspase-dependent apoptotic cells. As expected, we observed dying nuclei after irradiation, with a peak after 1 week and a decrease over time, but dying cells were still visible even after 6 months, suggesting a slow but constant loss of cells after photon irradiation (Fig. 3a and c). Interestingly, when we added a dephosphorylation step prior to TUNEL to unmask the 3’-P DNA ends, we detected a greater number of dying nuclei [16] (Fig. 3b and d) at every time point, suggesting that the mechanisms involved in irradiation-mediated cell death imply not only caspase-dependent apoptosis but also caspase-independent apoptosis and possibly necroptosis. It is also important to note that dying nuclei were localized almost exclusively in the outer nuclear layer (ONL), which is consistent with the significant and progressive decrease in the nuclear density of this layer at 1 and 6 months (Fig. 3e). Occasionally, some ganglion cells were stained with TUNEL-deP at 6 months. The fact that cell death was observed at early and later time points after irradiation suggests that it could result from continuous and unregulated stress occurring within the retina.

### Glial cells activated after irradiation in the retina contribute to vascular damage

**Fig. 4:**
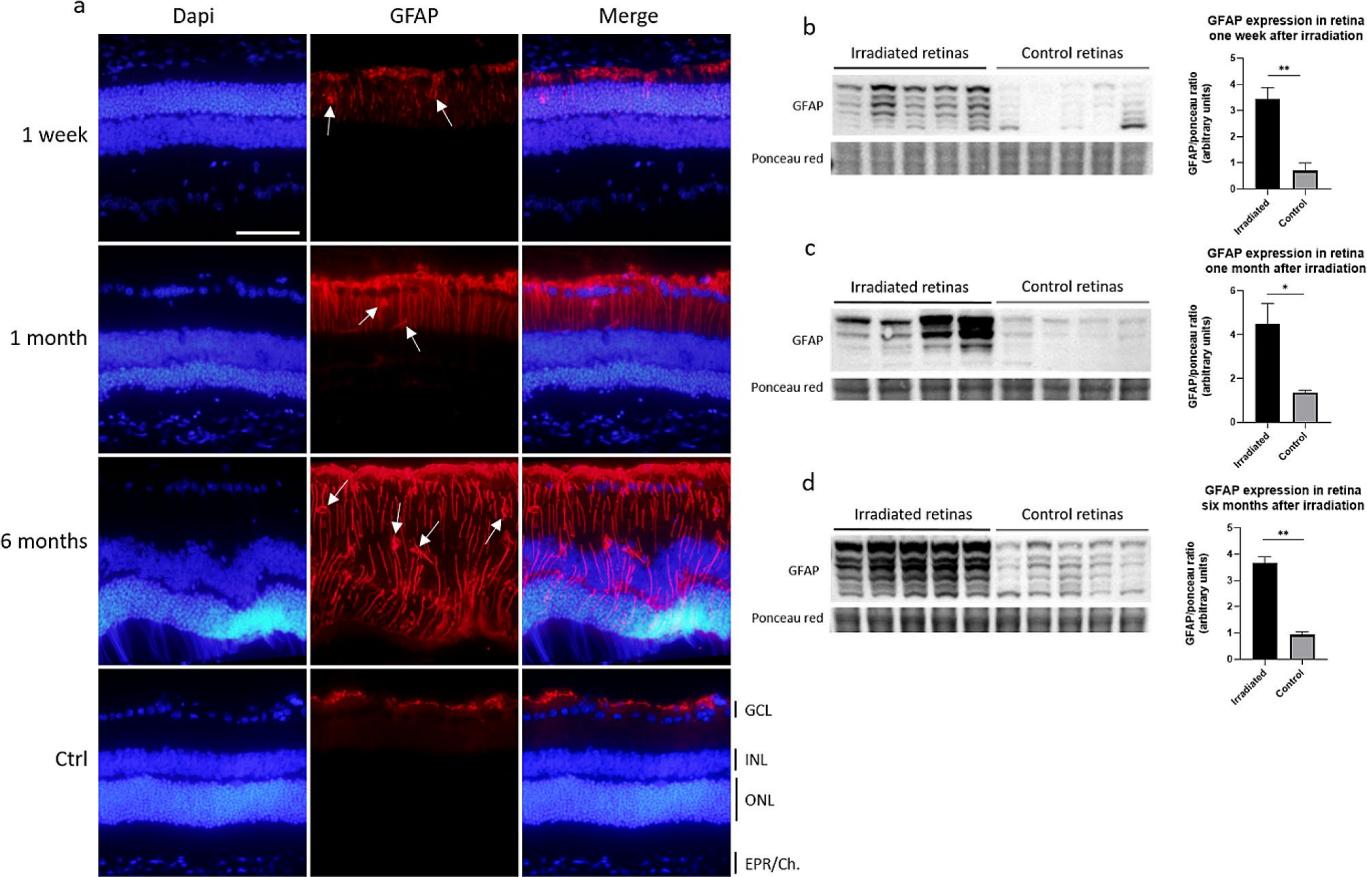
Photon irradiation induced GFAP overexpression in the retina. (**a**) GFAP expression (in red) in Müller cells and microglial cells (white arrows) on cryosections of irradiated eyes after 1 week, 1 month, 6 months and on non-irradiated eyes used as control (Ctrl). Nuclei are stained with Dapi in blue. The scale bar represents 50 μm. GCL means ganglion cells layer; INL, inner nuclear layer; ONL, outer nuclear layer; RPE/Ch, Retinal Pigment Epithelium/Choroid. (b to d) Western blots of irradiated and control retinas after 1 week (**b**), 1 month (**c**), 6 months (**d**) and their quantification. The integrated density of the several GFAP specific bands was measured and normalized over the ponceau red staining. *n* = 4 (M1) or *n* = 5 (W1, M6), Mann-Whitney statistical test. *p < 0.05; **p < 0.005

Glial fibrillary acidic protein (GFAP) is a marker of gliosis due to the inflammatory response in the retina. In control retinas, GFAP is expressed by microglia such as astrocytes, as well as by the feet of Müller cells, both of which are located in the ganglion cell layer (GCL) (Fig. 4a). After irradiation, we observed a progressive increase in GFAP expression in the Müller cell body through the distinctive feature of vertical filaments from the GCL to the external limiting membrane, which was clearly visible 6 months after irradiation. As soon as a week after irradiation and throughout the follow-up period, GFAP-positive cells, which could correspond to astrocytes or microglia, migrated from the most inner retinal layers toward the internal plexiform and internal nuclear layers (Fig. 4a, white arrows). The GFAP protein expression rise was validated by Western blot analysis of retinas and was significant from 1 week to 6 months post irradiation compared with that in non-irradiated eyes (Fig. 4b to d). These results suggested that the glial inflammatory response appeared quickly after irradiation and seemed to be amplified with time up to 6 months after photon beam treatment.

**Fig. 5:**
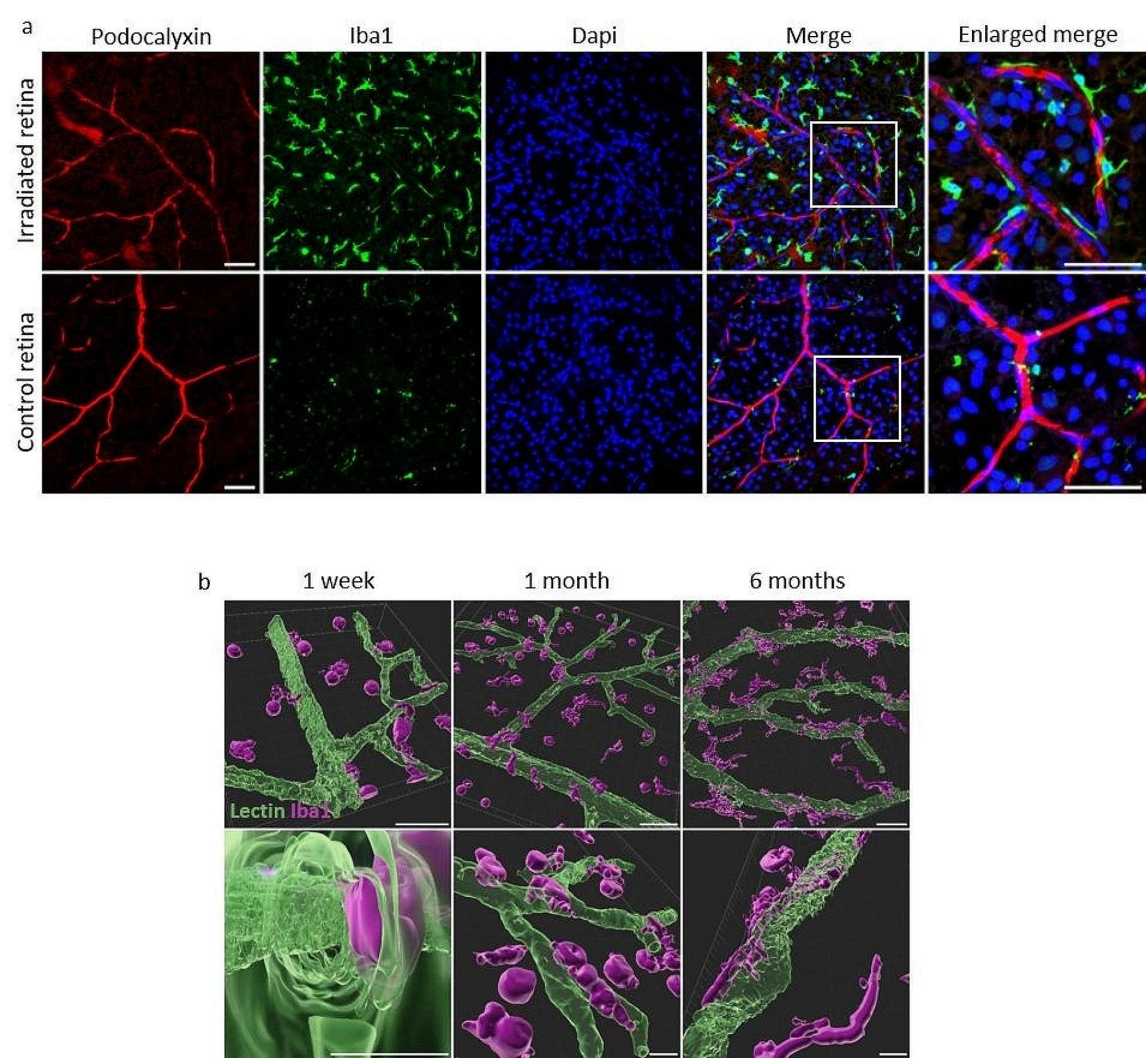
Microglia in contact with retinal vessels. (**a**) Retinal vessels and microglia invasion 6 months after irradiation. Retina flat mounts stained with anti-podocalyxin (in red) to visualize luminal membrane of endothelial cells, anti-Iba1 (in green) to localize microglial cells or macrophages and Dapi (in blue) to stain nuclei. Scale bars represent 50 μm. (**b**) 3D-reconstruction of microglia invasion. Images from retina flat mounts stained with lectin (in green) to show vessel walls and anti-Iba1 (purple) to visualize microglial cells and macrophages. Scale bars represent 20 μm on the upper row, and 5 μm on the bottom row. Confocal microscopy images processed with IMARIS, Oxford Instrument, UK.

Microglial migration in the retina seemed to start simultaneously with Müller cell activation. To decipher the inflammatory cellular response to irradiation, we stained retinal flat mounts with other types of microglial markers, such as Iba1 (Ionized calcium-binding adaptor molecule 1), a pan microglia and macrophage marker that does not label neurons or astrocytes [18]. As illustrated on Fig. 5a, 6 months after irradiation, the entire retina was invaded by microglial cells stained with Iba1, while a 6-month control retina showed only few microglial cells. It is interesting to note that numerous cells were located close to the vessels, whose luminal membrane are stained with podocalyxin [19]. Microglial ramifications seemed to be directly in contact with endothelial cells (Fig. 5a, magnified area), and in some cases, vessels were wrapped by those inflammatory cells. Noticeably, the vessels appeared altered with a loss of podocalyxin staining in comparison to those in non-irradiated retinas, with focal lumen constriction showing progressive vessel alterations, possibly mediated by the microglial cells. Tridimensional reconstructions support these observations (Fig. 5b). At one week after irradiation, Iba1-positive cells were mostly rounded and distant from the vessels. Few ones harbored extensions that scratched the vessel and crossed it. One month after irradiation, the Iba1-positive cell density increased, and the morphology of the cells changed. They appeared more branched and surrounded the vessels. At 6 months, almost all cells were spread out on the vessel with many ramifications, suggesting a role in post-radiation microvascular changes.

### Outer blood–retinal barrier disruption contributes to retinal inflammation

**Fig. 6:**
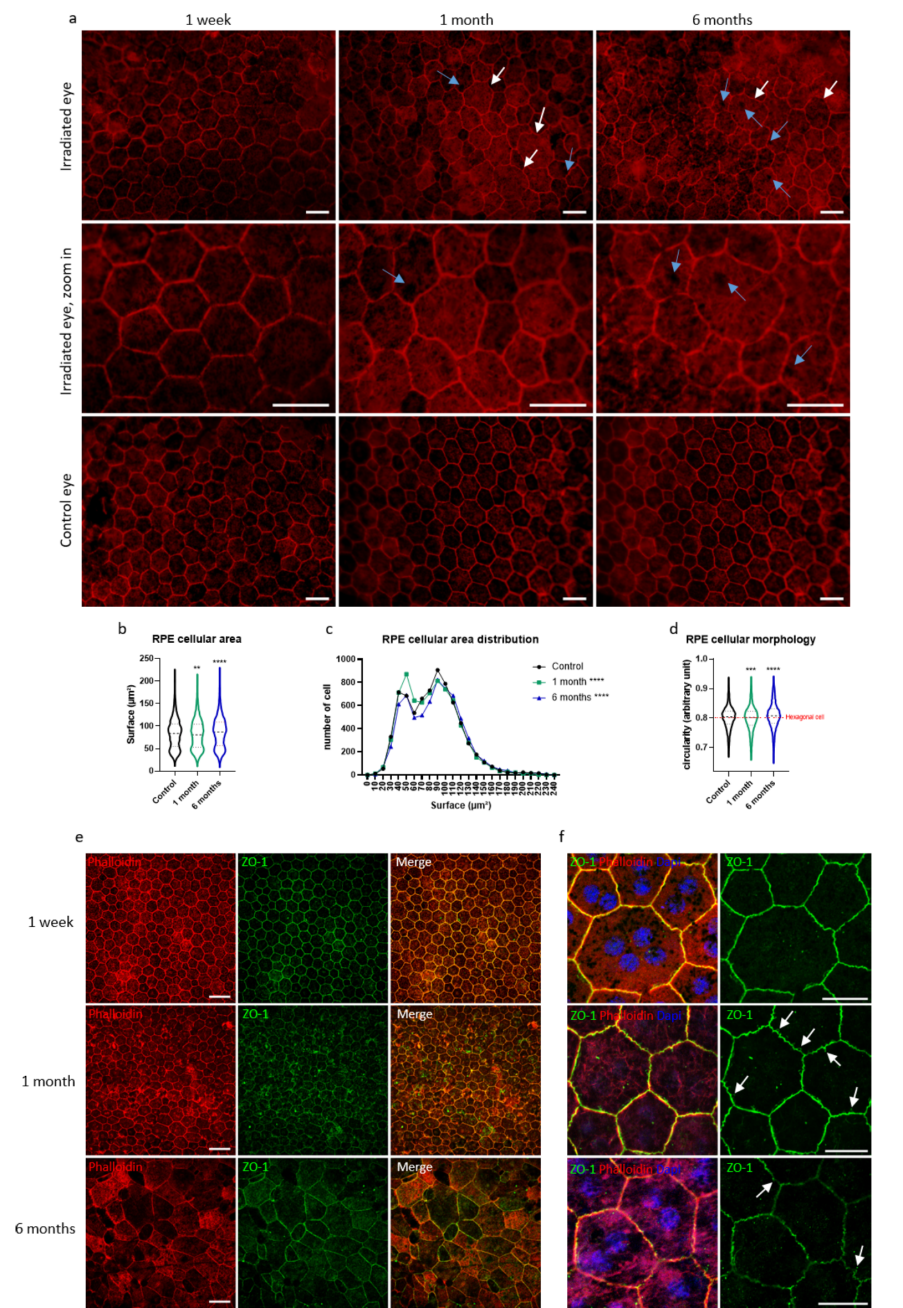
RPE progressive disorganization after irradiation. (**a**) RPE/Choroid complex flat mounts stained with Phalloidin (in red) to visualize actin-F cytoskeleton. After a week, almost no changes were visible on the RPE integrity. After a month, large rounded cells (white arrows) and holes (blue arrows) within cells were observed. (**b**) Quantification of RPE cell surface. One-way ANOVA statistical test, followed by a Dunnett’s multiple comparison test. (**c**) Distribution of the cell area of RPE cells. The statistical analysis was performed using a Chi-square test comparing the distribution of the control (expected) with the distribution of the 1 month or 6 months (observed). (**d**) Evaluation of the RPE morphology according to their circularity. Hexagonal cells like RPE cells have a circularity of 0.8. The more cells become round shaped, the more their circularity tends towards 1. One-way ANOVA statistical test, followed by a Dunnett’s multiple comparison test. For all statistics, *n* = 4 eyes and between 1365 and 2360 cells per eye have been measured on 18 images representative of the whole RPE. **p < 0.005; ***p < 0.0005; ****p < 0.0001. For the control, 1-month and 6-months group, the mean circularity was 0,8012; 0,7985 and 0,8079 respectively with a standard deviation of 0,03860; 0,04185 and 0,04328. (**e**) RPE flat mounts stained with phalloidin (in red) and ZO-1 (in green) to localize tight-junctions, one week, one month and 6 months after irradiation. Scale bars represent 50 μm. (**f**) Enlargement of ZO-1 staining on RPE flat mounts, associated with the merge image with phalloidin (in red), ZO-1 (in green) and Dapi (in blue). Abnormalities are enlightened with white arrows. Scale bars represent 20 μm

The blood–retinal barrier (BRB) is formed by tight junctions between endothelial cells of the retinal vasculature (inner BRB) and by those between retinal pigment epithelium (RPE) cells (outer BRB) [20]. After exploring the cellular changes associated with inner BRB alterations after irradiation, we investigated possible changes in the outer BRB. One week after irradiation, we did not observe major alterations in the structure of the RPE, which was stained with phalloidin to visualize the F-actin cytoskeleton (Fig. 6a), despite the small number of missing cells. However, one month after photon beam irradiation, morphological alterations became visible with modifications in cellular shape and size: some epithelial cells were no longer hexagonal but had a more rounded or irregular shape. We also observed stress fibers in most of the cells, as well as some holes inside the cells, with no surrounding actin belt. Some openings of the actin cytoskeleton barrier were visible, and the RPE cells were no longer in contact with each other.

After 6 months, all the previous alterations were still visible, with a large number of intracellular holes and an important proportion of RPE cells exhibiting altered morphology (Fig. 6a). Analysis of the morphological parameters of the RPE cells revealed significant changes in the global cellular area and in the cellular area distribution at 1 and 6 months post irradiation compared to control RPE from non-irradiated eyes. (Fig. 6b and c). Likewise, the RPE cell morphology changed after irradiation, becoming less angular than those in the controls (Fig. 6d).

The outer BRB is formed by tight junctions between RPE cells, involving several proteins, including Zonula occludens-l (ZO-1). ZO-1 localization did not seem to be altered one week or one month after irradiation (Fig. 6e). However, the ‘zigzag’ patterns might suggest a contraction of the cellular membrane (Fig. 6f). Several abnormalities were observed at one month, with loops, holes and sometimes disruptions in the ZO-1 pattern. At late stages, ZO-1 expression was reduced and even absent from some RPE cells in damaged areas (Fig. 6e). Alterations in tight junctions, probably related to actin cytoskeleton remodeling, in addition to missing cells and intracellular holes, could be responsible for outer BRB leakage.

**Fig. 7:**
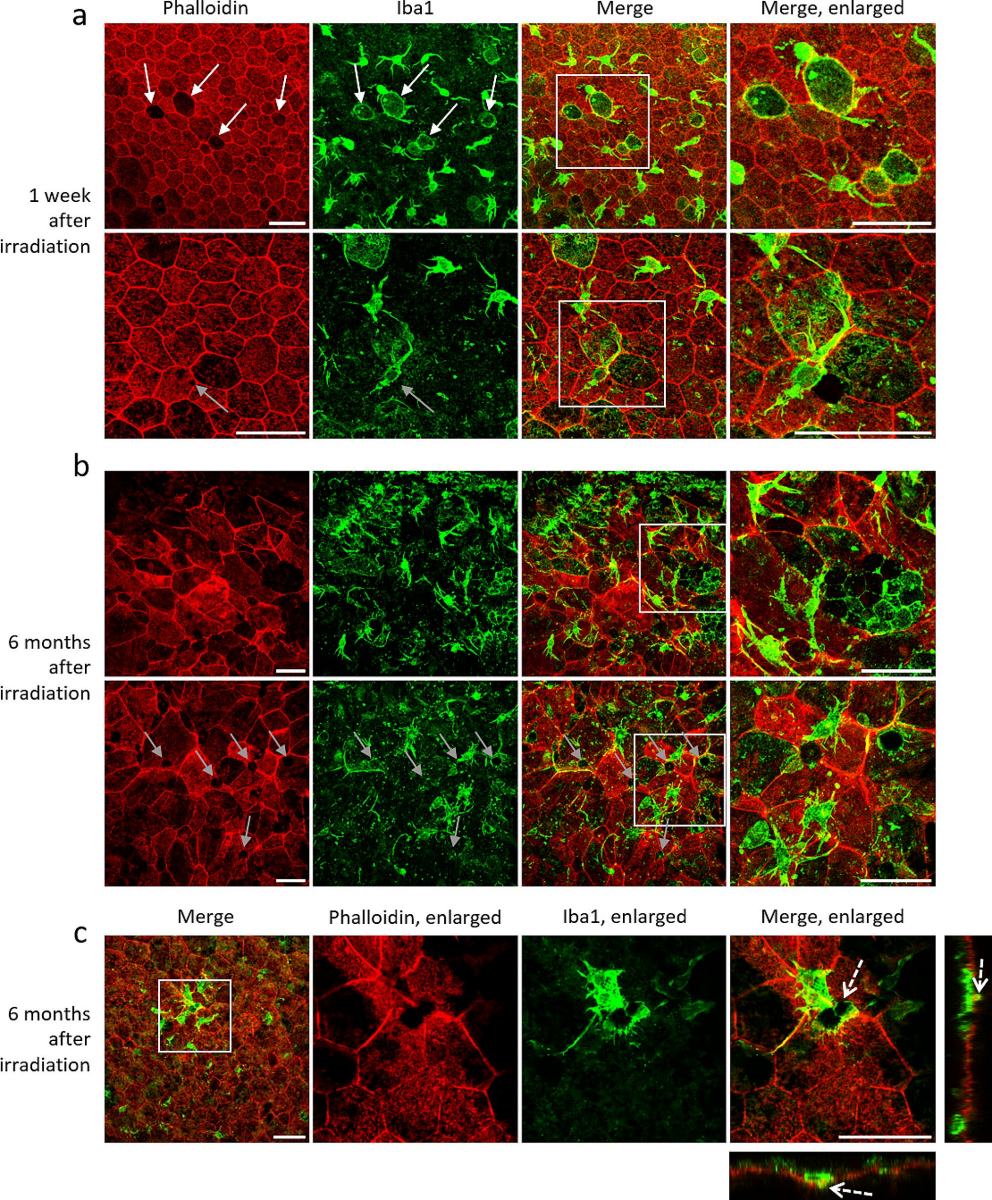
Microglia invasion of RPE after irradiation. RPE/Choroid complex flat mounts stained with Phalloidin (in red) allowing the visualization of F-actin and Iba1 (in green) to identify microglial cells, observed 1 week (**a**) and 6 months (**b**) after photon irradiation. Scale bars represent 50 μm. White arrows show missing RPE cells replaced by Iba1-positive cells. Grey arrows indicate holes inside or in between RPE cells. (**c**) After 6 months, many holes are visible in the RPE layer, surrounded by one or several Iba1-stained cells going through, see dotted arrows on X and Y projection. Scale bars represent 50 μm

To determine the role of the holes observed in the RPE after irradiation, we performed staining for several microglia, such as Iba1 for microglia or CD68, used as pan-macrophages marker. One week after irradiation, although the global structure was maintained, we locally found missing cells and intracellular holes (Fig. 7a). Surprisingly, we found microglial cells at the exact location where RPE cells were missing, and the shape of the Iba1-positive cells perfectly fitted with the previous hexagonal cells (Fig. 7a enlarged). In addition, one or several dendritic microglial cells were found close to or around every intracellular hole (Fig. 7a enlarged). Finally, as soon as a week after irradiation, the apical surface of the RPE was covered with numerous dendritic cells, and some were also found under the RPE in the choroid layer. Six months after irradiation, we observed the same dendritic cells on the RPE surface, which were often grouped as patches. The same observation of Iba1-positive cells was made, as shown in Fig. 7b, where several microglial cells were found in a large hole in the RPE, and some of them also presented an RPE-like hexagonal shape (Fig. 7b enlarged). As observed after one week, every intracellular hole was surrounded by one or several Iba1-positive cells after 6 months. These dendritic cells were crossing the RPE layer through the observed intracellular holes. Hence, as visible on Fig. 7c, a dendrite marked by Iba1 was clearly located under the basal membrane of the RPE cells (Fig. 7c, dotted arrows on the X and Y projections), while the cellular body was at the level of the RPE. In various cases, we observed that cellular prolongation of Iba1-positive cells ran along the RPE membrane. Finally, microglia were also observed under the RPE in the choroid layer. Due to the poor condition of the RPE at 6 months after irradiation, quantification of Iba1-positive cells on flat mounts was not possible. However, we counted Iba1-stained cells in the retina on cryosections and found that the Iba1-positive cell population significantly increased after irradiation, starting as soon as one week and remaining present after 6 months.

**Fig. 8:**
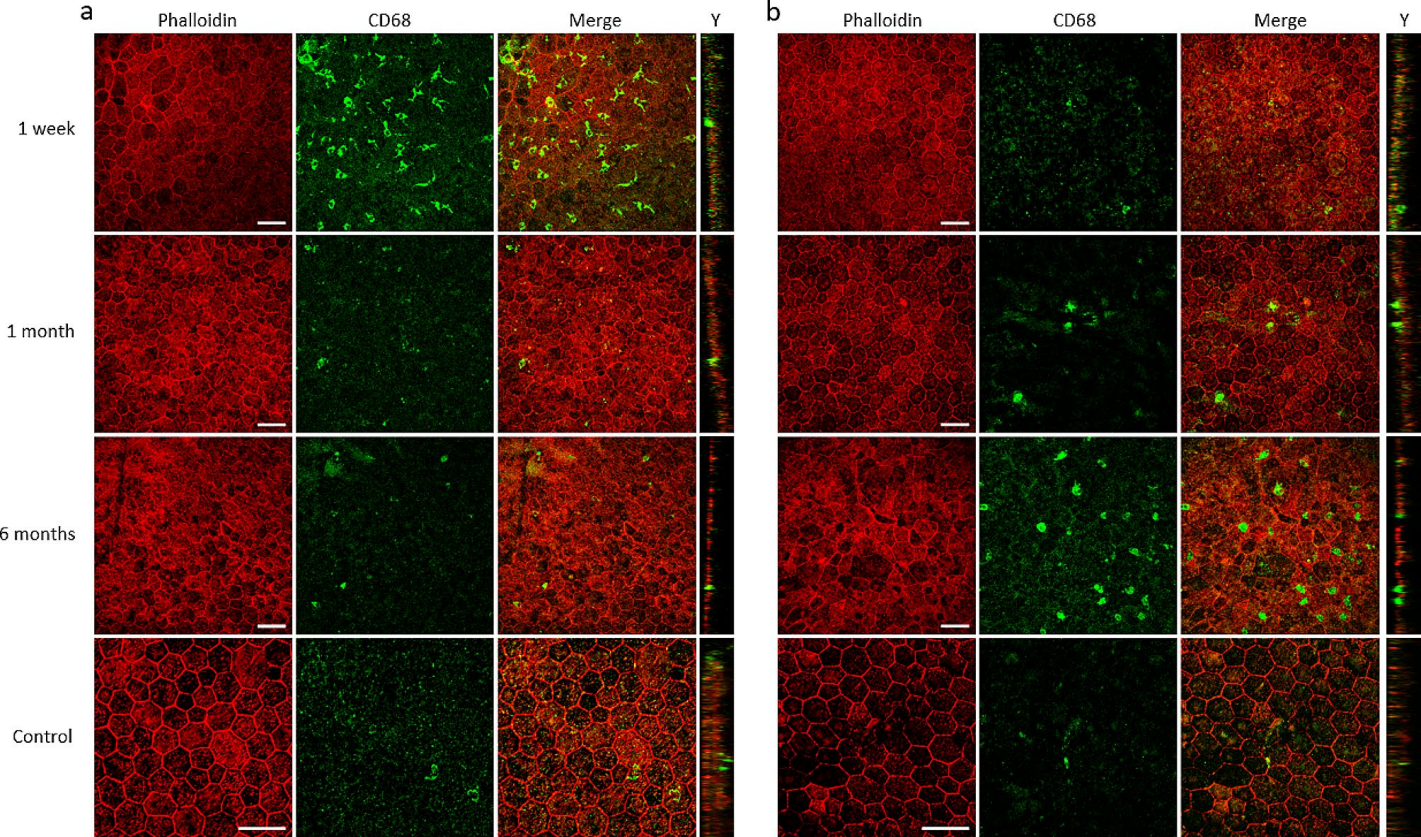
Macrophage distribution on RPE after irradiation. RPE/Choroid complex flat mounts stained with Phalloidin (in red) allowing the visualization of F-actin and CD68 (in green) to identify mature macrophages, observed 1 week, 1 month and 6 months after photon irradiation. Non-irradiated eyes were used as control. (**a**) Central RPE. (**b**) Peripheral RPE. Scale bars represent 50 μm. Images are Z-projections of stack images. Y-images for each panel represent the Y-projection of the corresponding stack

We also examined macrophages on RPE flat mounts and found that CD68-stained macrophages were present on the RPE as soon as one week after irradiation (Fig. 8a), mostly in the central area of the epithelium. They looked star-shaped, and their presence decreased after one month. Few cells were visible at 6 months, similarly to control RPE. However, these macrophages were mostly on the apical surface of the RPE after irradiation, while they were maintained in the choroid layer in control eyes (Fig. 8a, Y projection). On the other hand, few CD68-positive cells were observed on the peripheral RPE one week after the photon beam (Fig. 8b). Their number increased slightly after one month, but they were abundant 6 months after irradiation. Unlike what was observed in the central RPE, those cells were more round-shaped than star-shaped. However, they were similarly distributed on the apical surface of the epithelium (Fig. 8b, Y projection).

## Discussion

Despite the development of several animal models of RR, the role of inflammation in the pathogenesis of RR remains unclear. Among those studies, several techniques were used to decipher the molecular mechanisms of RR, with a special focus on the damage occurring in the retinal vascular and structural alterations, but the heterogeneity of irradiation protocols (irradiated structure, doses, fractions, and timing of in vivo experiments) makes the understanding of the kinetics of events challenging [6,21–25]. Here, we characterized the kinetics of retinal and RPE/choroidal damage induced in a photon beam radiation model. We highlighted vascular damage and inner and outer BRB alterations leading to a long-lasting inflammatory response and loss of photoreceptors. We chose to irradiate rats at a dose of 45 Gy delivered in 3 fractions, which was considered comparable to the average dose delivered clinically to patients affected by ocular tumors (30 Gy for choroidal metastases, 45 Gy for retinoblastoma and 60 Gy for uveal or conjunctival melanomas). Photon beams have been used due to their availability in a research setting with SARRP, but RR occurs after irradiation by all types of sources (proton therapy, photon X-rays, iodine or ruthenium plaque brachytherapy), suggesting common cellular underlying mechanisms [3]. We chose 3 time points to explore cellular and molecular mechanisms: 1 week and 1 month, corresponding to early stages, and 6 months, corresponding to later stage. Due to its small size, the whole rat eyeball was irradiated, including the lens, which protrudes into the posterior segment. As this leads to the development of a premature cataract, in vivo follow-up of the retinal vasculature and structure with angiography and OCT after photon beam became impossible as soon as 3 to 4 weeks following treatment. However, the vascular network was already modified a week after irradiation, with an increase in the surface area occupied by small vessels, suggesting the dilation of small capillaries as a first response to the photon beam irradiation. This phenomenon seemed to be transient since 1 and 6 months after irradiation, when the surface occupied by small vessels and capillaries was significantly decreased compared to control. These results are consistent with previous studies showing a reduction in retinal capillary diameter or vascular loss after irradiation at lower doses [23,24]. The loss of capillaries is due to the loss of endothelial cells through several cell death waves [26]. This is accompanied by global retinal hypoxia at 6 months, which is in accordance with the reduction in retinal capillary diameter. Surprisingly, it is also accompanied by an increase in the surface area occupied by larger vessels, as previously described in several studies [22,26]. This finding might be explained by the fact that as affected capillaries become occluded, the blood flow is diverted into nearby vessels, leading to their dilation under the hemodynamic load, as hypothesized by Archer et al. Moreover, the increased blood flow in damaged vessels could explain the formation of microaneurysms and capillary abnormalities, such as blebs, observed here 6 months after irradiation. Membrane blebs have also been observed in a diabetic retinopathy model [27], in which the Rho-associated kinase (ROCK) pathway has been shown to play a role in vascular constriction and microvascular occlusion [17]. This hypothesis should be further studied in our RR model.

Several studies on both rat and mice models of RR have described a first peak of cell death with apoptotic cells detected in all retinal layers as early as 12 h after a lower dose of irradiation [22]. This early cell loss is possibly due to direct radiation damage to DNA, membrane lipids and proteins, which causes apoptosis through the p53 pathway [28,29]. A second wave of delayed cell death resulting from oxidative damage mediated by reactive oxygen species (ROS) produced subsequently in irradiated tissue [30] can induce different types of cell death [31] until one year after irradiation [32]. Chronic hypoxia resulting from vascular damage can cause inner retinal cell loss [33] but might not induce direct photoreceptor cell death since hypoxia-induced factor 1 (HIF-1) is rather protective for photoreceptors [34], and the oxygen supply for photoreceptors mostly relies on the choroidal circulation. On the other hand, oxidative stress and persistent inflammation [35,36], which are mediated by micro- and macroglia but also by macrophages originating from the choroid, can cause photoreceptor cell death. In our model, after the expected peak of cell death observed at one week, photoreceptor cell death was detected at one and six months, and was two times greater with a modified protocol of TUNEL staining [16] than with the regular protocol, indicating that both caspase-dependent cell death and caspase-independent apoptosis, as well as possibly necrosis or necroptosis [37,38], occur along with signs of outer retinal inflammation. However, the involvement of these cell death pathways remains to be elucidated with a dedicated study.

The progressive but constant loss of photoreceptors, leading to reduction of nuclear density, but with only a slightly reduction of the ONL thickness suggests the presence of retinal edema, witnessing retinal inflammation. Retinal edema is a common feature in patients with RR but also in several retinal disorders, such as diabetic retinopathy, where the role of microglial and Müller cells is well established [39]. Müller cells maintain the homeostasis of the retina and can be activated by numerous stimuli. Reactive Müller cells are first neuroprotective but in the case of homeostatic imbalance, they can contribute to neuronal degeneration, retinal edema [11], and photoreceptor cell death [39]. In our model, Müller cells were activated as soon as 1 week after irradiation, as indicated by an increase in GFAP expression, which continued to increase progressively until 6 months where the entire Müller cells body were GFAP positive. This progressive and persistent inflammatory state might contribute to retinal degeneration and vasculature alterations induced by irradiation. It has been shown in a diabetic retinopathy model that GFAP overexpression is also an early feature of glial reactivity leading to morphological and structural changes, such as overexpression of the water transport channel aquaporin 4 inducing retinal edema [40], secretion of cytokines and inflammatory factors damaging the BRB [41], and production of VEGF that in turn increases capillary permeability, vascular leakage and promotes neovascularization [42]. Targeting specifically Müller cells is a promising therapeutic strategy for retinal neuroprotection [43] as they could directly influence microglial activation and migratory mobilization [44]. GFAP is overexpressed not only by Müller cells in cases of retinal inflammation but also by resident microglia and astrocytes [45]. Here, we observed that GFAP-positive dendritic-shaped cells migrated toward the outer retina, reaching the ONL, suggesting that retinal inflammation maintained by Müller cell gliosis is accompanied by retinal invasion of astrocytes and/or microglial cells. This progressive inflammation that persists at late stages might contribute not only to retinal degeneration but also to microvascular alterations after irradiation.

The presence of a retinal inflammatory state is also confirmed by the increase of Iba1-expressing cell density in the retina beginning in the early stages, from one week after irradiation until 6 months, when microglia are found in every layer of the retina and on the apical surface of the RPE. Resident microglia are physiologically found mostly in the GCL but also in the inner and outer plexiform layers (IPL and OPL) [46,47]. However, here, the density of microglial cells was increased compared to control retinas. Interestingly, at 6 months, most microglial cells were close to or in contact with vessels, and some of them were even wrapped around the vessels. Microglia are increased in areas of retinal hypoxia and neovascularization where they aggregate in and around neovascular tufts on the inner surface of the retina [48,49]. Specific markers were used to establish that they were activated into macrophages. Indeed, these inflammatory cells can undergo phenotypic and functional modifications to become macrophages, which are able to release inflammatory cytokines to recruit inflammatory effector cells and thus exacerbate inflammation [50]. Moreover, there are several types of macrophages, depending on their anti-inflammatory (M2), pro-inflammatory (M1), or perivascular characteristics [47,50,51]. The latter, also called vessel-associated microglia, are involved in vessel maintenance and repair as well as in the clearance of infiltrating cells of the neuro-glial-vascular unit [52], which could be relevant in our RR where vessels are altered. Interestingly, in diabetic retinopathy, reactive microglial cells are associated with the perivascular unit. They are also hypothesized to exacerbate vascular permeability and propagate the inflammatory response [53]. In RR, endothelial cells are also affected by irradiation, as described in several previous rodent models^23,26,]^ and these alterations have also been established in our model as described above.

Our results showed that irradiation also exerts deleterious effects on the outer blood‒retinal barrier formed by tight junctions between RPE cells [54,55], which can modify the photoreceptor microenvironment and contribute to outer retinal edema and photoreceptor cell death. After irradiation, the epithelium is rapidly affected, with some RPE cells missing and with intracellular holes leading to potential outer BRB breakdown. The number of these features increased over time, which led to a deteriorated epithelium 6 months after irradiation. This was accompanied by a global change in the surface and shape of RPE cells, junctions between cells were locally opened, cells displayed an increased range of cellular area with an increasing proportion of small cells, even though larger multinucleated syncytia-like cells were also detected. Their hexagonality was also lost in favor of round-shaped cells. These alterations were similar to those detected in the RPE of several models: Goto Kakizaki rats, used as a model for diabetic retinopathy [17,56]; OXYS rats, used as an AMD-like retinopathy model [57]; and light-induced retinal degeneration murine models [58]. However, the pores or transcellular holes in the RPE remain poorly described in the literature. In streptozotocin-induced diabetic rats, small holes have been described only between cells, resulting in junction breakdown [59], which is clearly visible through disrupted staining of ZO-1 and is a commonly used marker of tight junctions. In our RR model, these junctions endured membrane tension, as suggested by the ‘zigzag’ pattern, which was visible as soon as one week after irradiation. Loops and small holes were also visible after one month, similar to those observed in the above-mentioned diseases. The tortuous ZO-1 pattern has been described as a reorganization of the peri-junctional cytoskeleton, with ZO-1 being one of the regulators of the cytoskeleton and cell shape at cell–cell contacts [60,61]. Modifications in both the expression and localization of ZO-1 induced by photon beam irradiation might play a role in the changes in cellular shape observed in our model.

Unlike small intercellular holes, larger transcellular pores have only been described in the Goto Kakizaki model, but they display an actin-F ring, supporting an active transcellular trafficking hypothesis involving PKCz (protein kinase C zeta) [62]. These pores could participate to physiological processes facilitating the passage of microglia and macrophages between the retina and choroid. This also seemed to be the case here since every hole was surrounded by, or contained, one or several Iba1-positive cells, with some passing through. As most of the microglia are localized on the apical surface of the epithelium, we could hypothesize that microglial cells were leaving the subretinal space toward the choroid. This finding is also consistent with the increase in microglial density and migration to the outer retina observed after irradiation. These microglia were likely activated since CD68-labeled macrophages were similarly observed on the apical surface of the RPE. However, they are not evenly distributed, as they appear ramified and close to the optic nerve at early stages, while they are more round-shaped and peripheral at later stages. Microglial morphology is important since it indicates the inflammatory status of microglia. The moderate but thick ramifications indicate that they are reactive, while the round or amoeboid shape indicates that they are phagocytic, suggesting a macrophage phenotype [50,63]. This finding is consistent with our observations: after irradiation, the microglial cells migrate toward the RPE, differentiate into macrophages upon arrival at the RPE, where they can phagocytose cellular debris from dead photoreceptors, RPE or other cells. Nonetheless, it is not excluded that monocytes-derived macrophages, coming from the general circulation and crossing the BRB could also be involved in radiation-induced retinopathy and photoreceptor cell death, as described in AMD and RPE injury models [64]. It would be interesting to investigate whether these inflammatory cells are anti-inflammatory M1 and/or pro-inflammatory M2 macrophages or whether there is a switch in the two populations between the early and later stages. Specific markers, such as CD86, iNOS, CD163 or CD206 65, could also be used.

The observation of microglial cells covering the entire body of a RPE cell which appeared to be missing, is surprising. Indeed, there were no nuclei, no phalloidin background or stress fibers, and no melanin pigments detected inside those cells. However, their shape was conserved, and it appeared that the body of an Iba1-positive cell filled the shape of the missing RPE cell. Microglial cells lying on the RPE have already been described in various ocular diseases, such as AMD with choroidal neovascularization [8,66], diabetic retinopathy [67] and RPE injury, but this type of cell shape has never been described so far. Iba1 cells can be recruited to the injured area, but the replacement of RPE cells remains obscure. We could hypothesize that exposure to radiation leads to the death of some RPE cells and that microglial recruitment to the site is a mechanism to preserve the outer BRB integrity. Microglial cells could also be attracted to impaired RPE cells to improve the phagocytosis of cellular debris and the rearrangement of the cells to rapidly recreate the epithelial barrier. It is important to note that we did not observe CD68-positive cells with this shape; only Iba1-positive microglia were observed. Previous studies have shown that recruited microglia express selective inflammatory mediators prior to any pathological sign of disease [68], which is consistent with our RR model in which microglia are detected at very early stages. This raises the question of whether the microglia proliferate once in the subretinal space or the cells are non-proliferative, and the recruitment is constant and possibly increasing, which would explain the accumulation of these cells in the outer retina. The role of microglia also seems to depend on the presence or absence of other types of inflammatory cells, such as infiltrating monocytes, as well as on the cytokines and inflammatory or anti-inflammatory factors they can produce [8]. As they can also produce VEGF in response to necrotic RPE to contribute to angiogenic development [68,69], it would be interesting to study angiogenic markers such as VEGF and angiopoietin 1 and 2 or their receptor Tie2 since vascular proliferative complications are common features observed in RR.

Whether irradiation has generated choroidopathy [70] and, more specifically, a progressive loss of choriocapillaris blood flow, as suggested by clinical imaging studies [71,72], and whether choroidal inflammation could contribute to progressive damage to the RPE and photoreceptor loss will require further exploration.

## Conclusion

This study highlights the deleterious effects of irradiation on the RPE and retina, particularly on inner and outer blood retinal barrier integrity. This finding demonstrates the paramount implication of progressive and long-lasting inflammatory mechanisms involving different types of cells. Homologies between this rodent model and clinical observations from irradiated patients seem to be relevant for investigating RR mechanisms. Further investigations are required to better characterize choroidal/retinal inflammatory mediators to try and target pathogenic inflammation and prevent irreversible retinal damage after ocular irradiation.

### Electronic supplementary material

Below is the link to the electronic supplementary material.

Supplementary Material 1

Supplementary Material 2

Supplementary Material 3

Supplementary Material 4

Supplementary Material 5

Supplementary Material 6

Supplementary Material 7

## Data Availability

The datasets supporting the conclusions of this article are available from the corresponding author upon reasonable request.

## Abbreviations

AMD: Age-related Macular Degeneration
BRB: Blood‒Retinal Barrier
ERG: Electroretinography
GCL: Ganglion Cell Layer
GFAP: Glial Fibrillary Acidic Protein
Iba1: Ionized calcium-binding adaptor molecule 1
INL: Inner Nuclear Layer
IPL: Inner Plexiform Layer
OCT: Optical Coherence Tomography
ONL: Outer Nuclear Layer
RPE: Retinal Pigment Epithelium
RR: Radiation Retinopathy
TUNEL: Terminal deoxynucleotidyl transferase dUTP nick end labeling
VEGF: Vascular Endothelial Growth Factor
ZO-1: Zonula Occludens-l
