# Supplementary figures and images for: Role of inflammation in a rat model of radiation retinopathy
Source: J Neuroinflammation. 2024 Jun 24;21:162. doi: 10.1186/s12974-024-03151-2 (PMC11197380; doi:10.1186/s12974-024-03151-2)

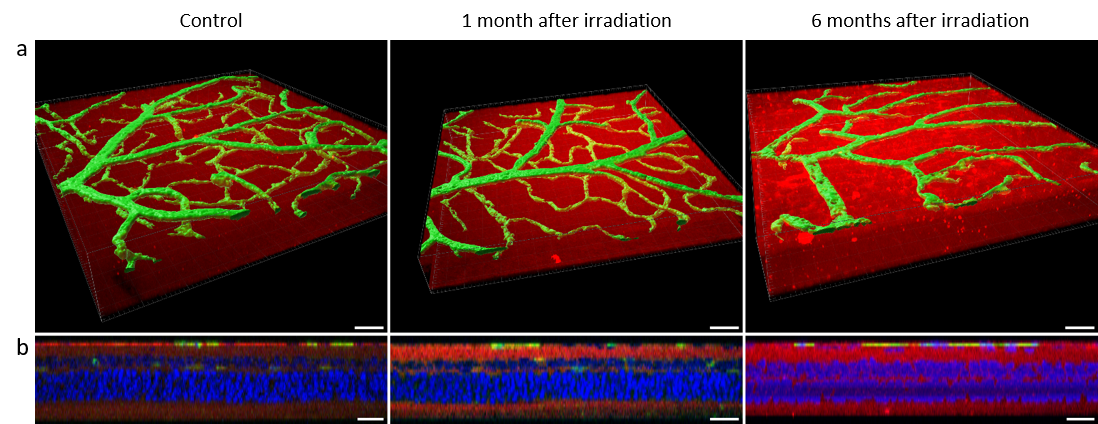

Supplement: Supplementary file 1 — Supplementary Material 1 [file 12974_2024_3151_MOESM1_ESM.tif]

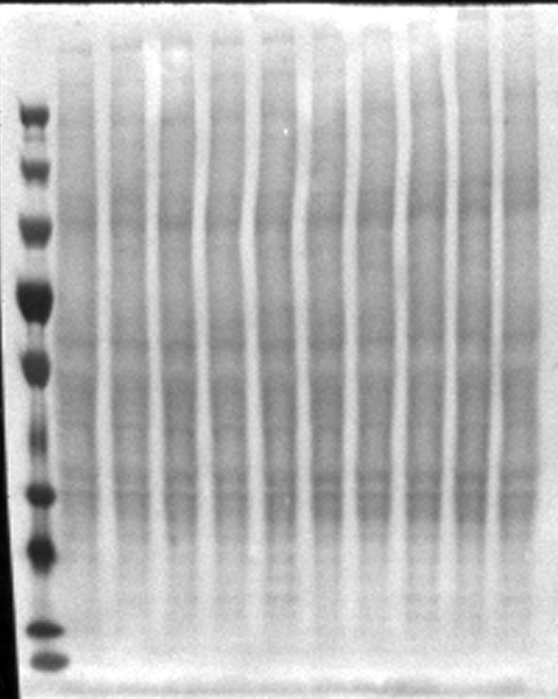

Supplement: Supplementary file 2 — Supplementary Material 2 [file 12974_2024_3151_MOESM2_ESM.tif]

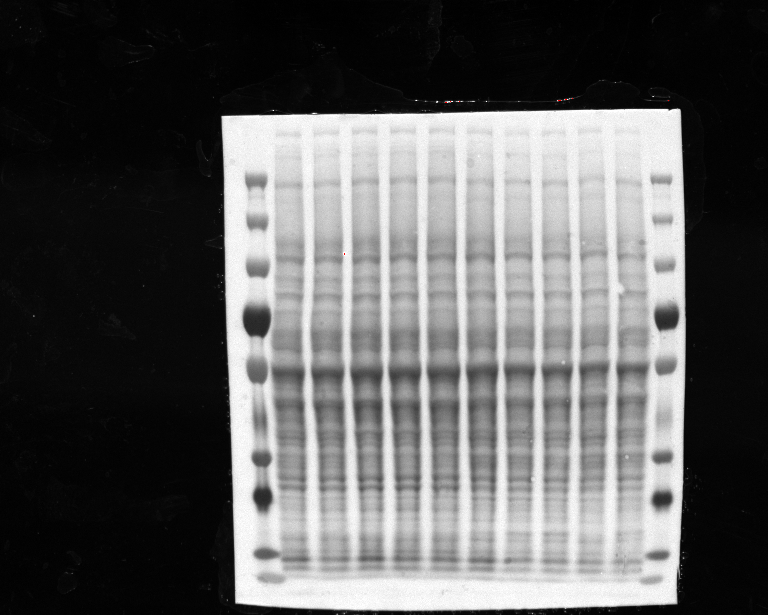

Supplement: Supplementary file 3 — Supplementary Material 3 [file 12974_2024_3151_MOESM3_ESM.tif]

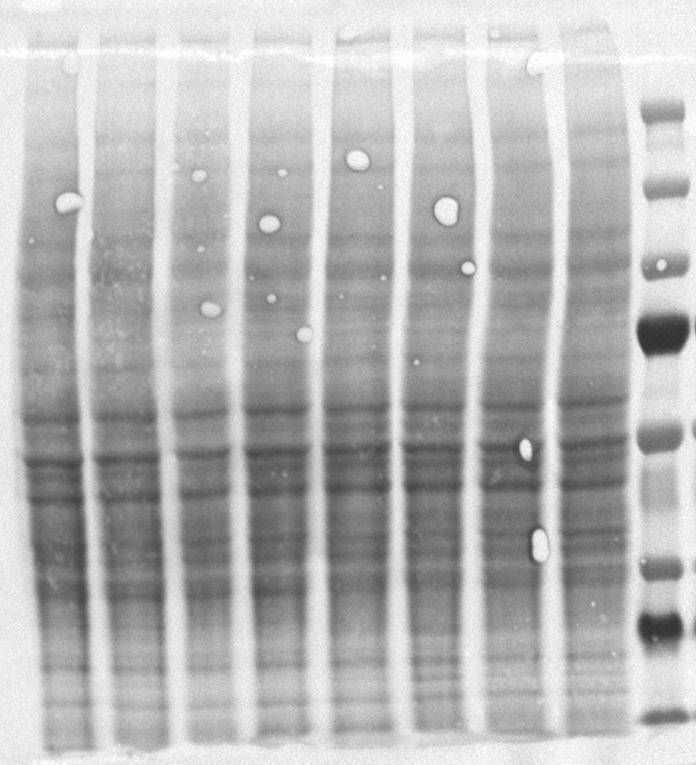

Supplement: Supplementary file 4 — Supplementary Material 4 [file 12974_2024_3151_MOESM4_ESM.tif]

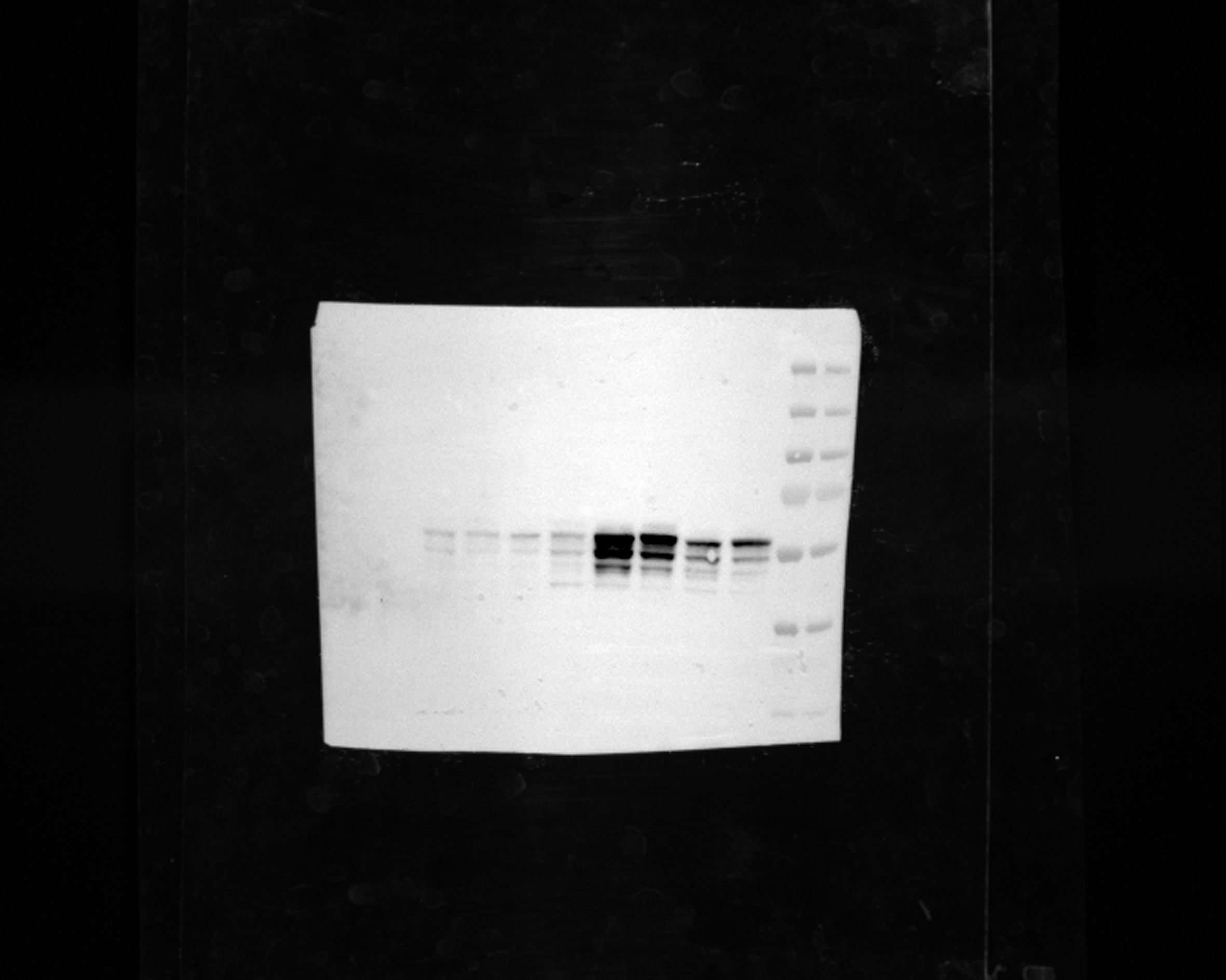

Supplement: Supplementary file 5 — Supplementary Material 5 [file 12974_2024_3151_MOESM5_ESM.tif]

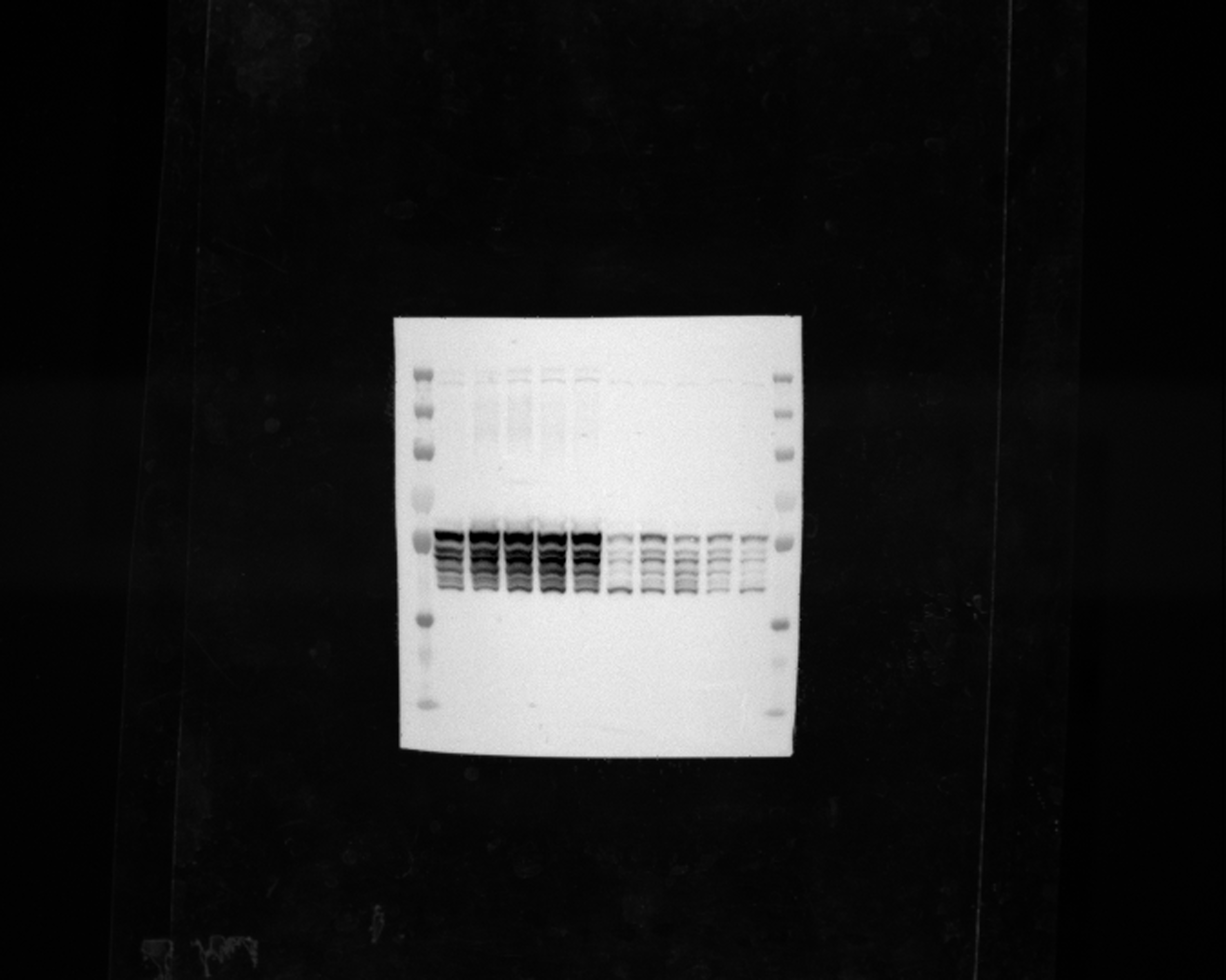

Supplement: Supplementary file 6 — Supplementary Material 6 [file 12974_2024_3151_MOESM6_ESM.tif]

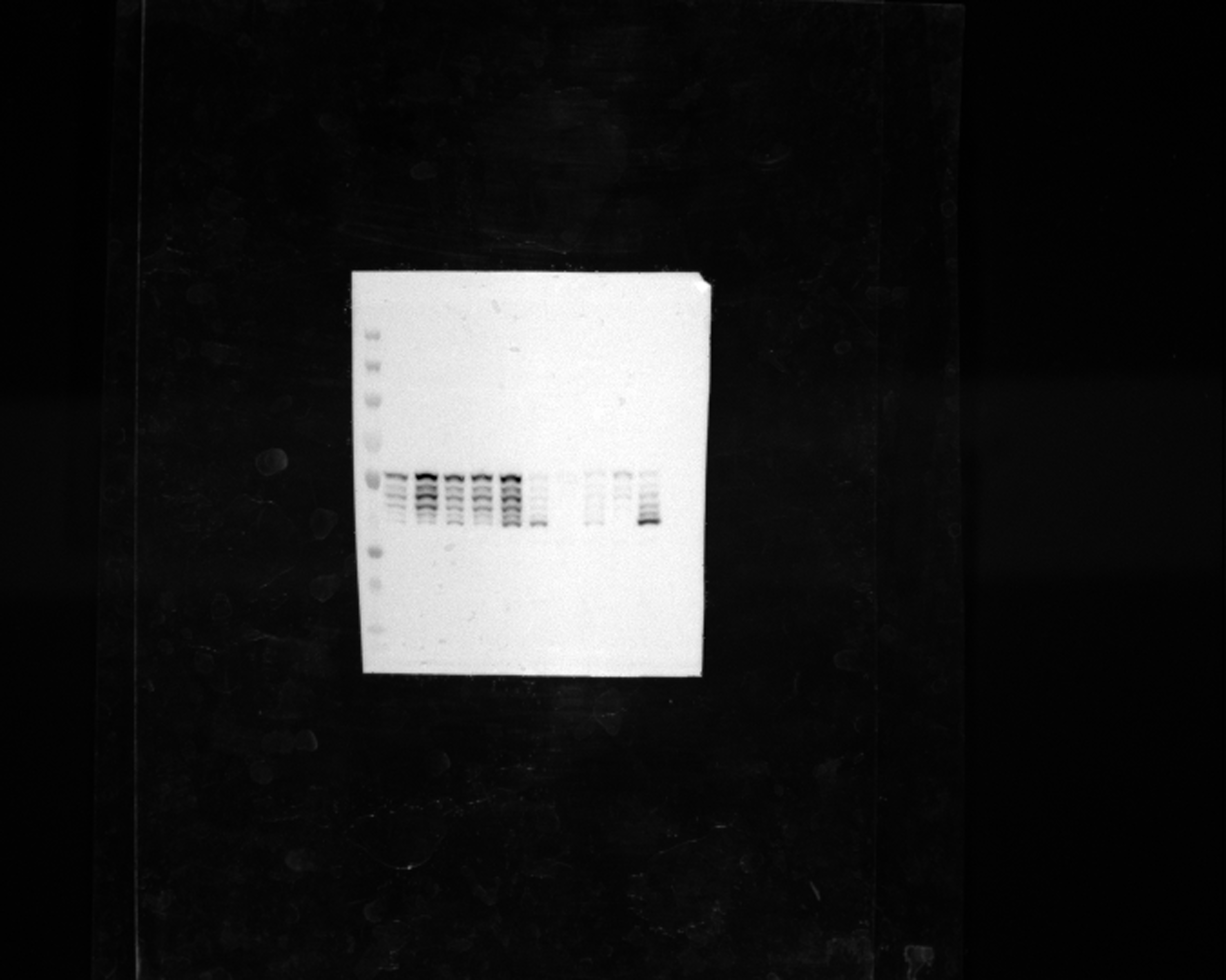

Supplement: Supplementary file 7 — Supplementary Material 7 [file 12974_2024_3151_MOESM7_ESM.tif]
